# The factor structure and subscale properties of the pain catastrophizing scale: are there differences in the distinctions?

**DOI:** 10.1097/PR9.0000000000000909

**Published:** 2021-03-17

**Authors:** Karon F. Cook, Sean Mackey, Corinne Jung, Beth D. Darnall

**Affiliations:** a Feral Scholars, Broaddus, TX, USA; b Department Anesthesiology, Perioperative and Pain Medicine, Stanford University School of Medicine, Palo Alto, CA, USA

**Keywords:** Pain catastrophizing, Psychometrics, Scaling, Self-report, Pain outcomes

## Abstract

Increasingly, studies have documented the negative impact of pain catastrophizing on health outcomes. The Pain Catastrophizing Scale (PCS) has been the measure of choice for many of these studies. The PCS provides 3 subscales for measuring pain catastrophizing: rumination, magnification, and helplessness. Factor analytic investigations of these factors have been limited by the sample size and relevance, and results have been inconsistent. No study has directly estimated the added value of subscale scoring of the PCS compared with scoring it as a single measure.

**Objective:**

The purpose of this study was to evaluate the dimensionality of PCS responses in a sample of patients with chronic pain (N = 8370).

**Methods:**

Data were randomly halved, and results were cross-validated. Both traditional factor analysis and traditional factor analyses were conducted.

**Results:**

Findings based on common factor analyses and on bifactor analyses supported the essential unidimensionality of PCS responses. In the bifactor analyses, the general factor accounted for 96% of the explained common variance in the modeling sample. After extracting the general factor, helplessness, magnification, and rumination subscales accounted for 7.0%, 0.0%, and 15%, respectively.

**Conclusion:**

The results do not necessarily disconfirm helplessness, magnification, and rumination as clinically meaningful theoretical distinctions. However, the PCS (at least as presently constructed) fails to discriminate these distinctions. Joint efforts in theory and measurement science could illuminate the role that posited “kinds” of pain catastrophizing play in individuals' pain experiences.

## Abstract 2

As presently constructed, the pain catastrophizing failed to discriminate effectively among levels of rumination, magnification, and helplessness in a sample of patients with chronic pain.

## 1. Introduction

In a 1983 study, Rosenstiel and Keefe identified 8 cognitive and behavioral strategies patients used to cope with back pain; one of which they named “pain catastrophizing.”^31^ Subsequently, Sullivan et al.^38^ expanded measurement of pain catastrophizing with the development of the Pain Catastrophizing Scale (PCS), a longer and more comprehensive measure of the construct.^38^ The PCS is widely used in international clinical care and research and has been translated into multiple languages.^37^ The measure targeted 3 subdomains that predominated research in the field—rumination, magnification, and helplessness. The initial analyses of PCS responses supported a 3-factor structure based on exploratory principal-components analysis of PCS item responses by 429 undergraduate psychology students.

Although some studies have supported a 3-factor structure of the PCS, some have not. Osman et al.^24^ attempted to replicate this factor structure in a sample of 288 undergraduate students including a subsample who were seeking care at a university health clinic. Their exploratory principal-components analysis supported a 2-factor rather than a 3-factor solution. When they specified 3 factors, however, their findings were similar to Sullivan et al's.^38^ In a follow-up study based on PCS responses of 215 community members and 60 pain outpatients, Osman et al.^23^ conducted a higher order latent factor analyses with rumination, helplessness, and magnification as second order factors. Their results strongly supported pain catastrophizing as a single construct comprised of the 3 hypothesized subdomains, a finding that has been replicated for English^11^ and non-English versions of the PCS^20,42^ and in a recent study comprised of 675 Chinese and non-Chinese patients with knee osteoarthritis.^22^ Further, in a study of the pediatric and parent versions of the PCS, Pielech et al.^25^ found no support for the 3-factor structure and concluded the responses to the items were unidimensional for both versions. These results raise doubt regarding the PCS's success in measuring 3 distinguishable domains of pain catastrophizing.

With the exception of the study by Ong et al.^22^ that used second-order factor analysis in a large clinical sample, previous studies either failed to use higher-order factor models or included few or no people with chronic pain (N = 60 in 1 study^23^; N = 146 in another, N = 0 in others).^11,24,38^ The purpose of the current study was to apply bifactor analyses to PCS item responses from a large clinical chronic pain population to evaluate the dimensional structure of the PCS and identify appropriate scoring strategies. Bifactor modeling was chosen over second-order factor analysis because the former provides estimation of the incremental reliability of subscale scores beyond that provided by the overarching general factor.

We undertook this study because of our interest in measuring patient outcomes with the least amount of response burden. Increasingly patient-reported outcomes are measured using item banks that have been calibrated to an item response theory (IRT) model^6,7,35^ and administered using computer adaptive testing (CAT).^33^ In CAT administrations, administration of items from IRT-calibrated item banks is tailored to individual respondents. This approach increases the ratio of measurement precision to response burden.^8^ However, the IRT models most commonly used for CAT require “essential unidimensionality”; that is, a large portion of the variance in item responses can be accounted for by a single, overarching domain.

## 2. Material and methods

### 2.1. Sample and design

Data are routinely collected from patients seeking care at the Stanford University Pain Management Center using the Collaborative Health Outcomes Information Registry (CHOIR), an open-source learning health care system platform.^36^ As a part of this learning health care system, patients completed a battery of surveys from the Patient-Reported Outcome Measurement Information System (PROMIS)^6^ and legacy measures including the PCS.^38^ The patient population is a heterogeneous mix of people with pain of various etiologies including neuropathic pain, musculoskeletal pain, headache, fibromyalgia, and a small minority of cancer pain. Data are collected at point of care. This study is a retrospective, methodological study of a subsample of N = 8369 CHOIR respondents.

### 2.2. Instrumentation

#### 2.2.1. Pain catastrophizing scale

The PCS is a 13-item, self-report measure of pain catastrophizing.^38^ Respondents are asked to rate the frequency of pain-related thoughts and feelings they have in response to actual or anticipated pain using a 5-point scale that ranges from 0 = not at all through 4 = all the time. The PCS was developed to be scored both as a total score to represent pain catastrophizing and as subscale scores representing rumination, magnification, and helplessness. The PCS is a widely used measure that has been translated from English in to many other languages.^5,20,34,39,42^ Scores on the PCS have been found to be responsive to changes in catastrophizing after intervention.^32^

#### 2.2.2. Demographics

Demographic variables are collected as part of the CHOIR. The deidentified data set for the current study, however, did not include these. We present demographics for a random sample of CHOIR data equal to the sample size of the study data (N = 8350). These data were used to characterize the sample with respect to sex, race, education, and age.

#### 2.2.3. Pain intensity

In CHOIR, patients report 7-day average pain intensity using a numerical rating scale (NRS) that ranges from 0 to 10. The NRS is commonly used to measure pain in chronic pain conditions and is highly correlated with other pain measures such as the visual analog scale.^40^

#### 2.2.4. Patient-reported outcome measurement information system measures

Included as part of the CHOIR survey are CAT administrations of PROMIS measures.^6^ To characterize the sample, we summarized scores for the sample on PROMIS measures of Pain Interference v1.0, Physical Function v1.0, Fatigue v1.0, Depression v1.0, Anxiety v1.0, Sleep Disturbance v1.0, Sleep Impairment v1.0, Anger v1.0, Emotional Support v2.0, Satisfaction with Roles and Activities v2.0, and Social Isolation v2.0. The PROMIS measures are scored on a T-score metric with a mean of 50 and a standard deviation of 10. The mean of 50 is calibrated to either the mean of a US reference sample that matched the 2000 General Census sample with respect to age, sex, race/ethnicity, and education or to a clinically relevant population.^13,17^ The PROMIS measures reported here were centered on the US general population representative sample. All PROMIS measures are scored such that higher scores indicate more of the symptom or function being measured.

### 2.3. Statistical analyses

Data were manipulated and descriptive analyses completed using IBM SPSS 25.^9^ Using the sampling option in SPSS, approximately 50% of the sample was selected to serve as the modeling sample (Sample_MODEL_). The unselected sample was used for cross-validation (Sample_X-VALID_). Exploratory factor analyses (EFAs), confirmatory factor analyses (CFAs), and bifactor analyses were performed separately on all subscales. All other analyses were conducted on the combined sample. Interitem consistency of subscales and of the combined items of the PCS was measured using Cronbach's alpha.

We conducted, on each sample, 2 EFAs—one extracted 1 factor, the other, 3 factors. Two CFAs were conducted. One CFA posited that a single dimension explained the variance of responses to PCS items (CFA_DIM=1_). The other posited that response variance could be explained by 3 factors, each represented by the subset of items intended to target helplessness, magnification, and rumination(CFA_DIM = 3_). An alternate hypothesis was modeled using a bifactor analysis.^29^ The bifactor model posited that variance in responses was explained by 4 factors—a general factor on which all items loaded and 3 orthogonal factors representing the unique variance accounted for by each of the 3 subscales. The bifactor and second-order factor models are similar but vary in the constraints imposed. Mansolf et al.^18^ published a detailed description and analysis of the distinctions between models.^18^ All factor models were completed using MPLUS version 8 and specifying a polychoric correlation to account for the categorical nature of the data.^21^ Factor loadings were estimated based on maximum likelihood and weighted least square mean and variance adjusted estimators and were rotated using PROMAX rotation, which allows factors to be correlated with each other.

The CFA and bifactor models were compared based on traditional fit statistics—comparative fit index (CFI), Tucker–Lewis index (TLI), root mean square error of approximation (RMSEA), and standardized root mean square residual and commonly applied criteria—CFI and TLI >0.95, RMSEA values <0.10, standardized root mean square residual values <0.08.^3,4,14,16,19,27^ For the bifactor model, we also calculated the omega-hierarchical (omega-H) statistic, which estimates the proportion of reliable variance associated with the general factor only, calculated as the ratio between general factor variance and total variance, including error.^28^ As such, it is a reliability statistic for the general factor. A criterion of omega-H > 0.8 has been recommended as a threshold for a measure's essential unidimensionality.^30^ Omega hierarchical subscale omega-H_SS_ estimates the reliability of subscale scores after variance from the general factor has been extracted. We also calculated explained common variance (ECV), which is the proportion of the modeled variance that is accounted for by the general factor.^30^ Explained common variance thresholds have been recommended for deciding on the value of subscale scores; ECV values below 0.70 suggest subscale scores have added value, whereas values above 0.90 suggest that they do not.^26^

## 3. Results

### 3.1. Sample characteristics

The total sample size was 8369; N = 4179 comprised Sample_MODEL_ and N = 4190 comprised Sample_X-VALID_. Table 1 summarizes the demographic characteristics and scale scores for the entire sample and also separately for Sample_MODEL_ and Sample_X-VALID_. The 2 samples were virtually equivalent regarding the evaluated variables, which was unsurprising given the sample sizes. The mean and standard deviation of the sample's ages were 49.1 and 16.1, respectively. Of those who reported sex (N = 8207), 67.1% were women and 32.9% men. Of those reporting race (N = 6453), the distribution was 61.3% White, 3.4% African American, 7.9% Asian, 1.1% Native American/Pacific Islander, and 26.3% others. A total of 5749 reported level of educational attainment—6.4% no high school, 7.7% high school, 61.7% college, and 23.5% graduate school.

**Table 1:** Mean, SD, minimum (min), and maximum (max) of self-report measures.

	Model sample N = 4179					Cross-validation sample N = 4190					Full sample N = 8369				
N	Min	Max	Mean	SD	N	Min	Max	Mean	SD	N	Min	Max	Mean	SD	
Pain catastrophizing full scale	4179	0	52	23.3	12.69	4190	0	52	23.3	12.68	8369	0	52	23.3	12.68
Helplessness subscale	4179	0	24	11.0	6.17	4190	0	24	10.9	6.20	8369	0	24	10.9	6.19
Magnification subscale	4179	0	12	4.0	3.03	4190	0	12	4.0	3.00	8369	0	12	4.0	3.00
Rumination subscale	4179	0	16	8.3	4.62	4190	0	16	8.3	4.62	8369	0	16	8.3	4.62
Pain intensity 7 d average visual analogue scale (0–10)	4179	0	10	5.5	2.21	4190	0	10	5.6	2.18	8369	0	10	5.6	2.19
PROMIS pain interference v1.0	4179	38	83	64.0	7.59	4190	38	83	64.0	7.49	8369	38	83	64.0	7.54
PROMIS physical function v1.0	2786	15	73	37.7	9.33	2869	15	73	37.5	9.32	5655	15	73	37.6	9.32
PROMIS fatigue v1.0	4179	24	84	58.6	10.03	4190	24	84	58.7	10.18	8369	24	84	58.7	10.11
PROMIS depression v1.0	4179	34	84	54.1	9.99	4190	34	84	54.0	9.93	8369	34	84	54.1	9.96
PROMIS anxiety v1.0	4178	32	84	55.2	9.95	4190	32	84	55.1	9.76	8369	32	84	55.2	9.85
PROMIS sleep disturbance v1.0	4178	26	83	56.2	9.33	4190	26	83	56.3	9.33	8369	26	83	56.3	9.33
PROMIS sleep impairment v1.0	4178	26	83	55.8	10.09	4190	26	83	56.2	10.03	8369	26	83	56.0	10.06
PROMIS anger v1.0	4177	28	85	49.7	10.43	4190	28	85	49.6	10.28	8368	28	85	49.6	10.35
PROMIS emotional support v2.0	4177	20	66	50.9	9.42	4190	22	68	42.4	9.78	8368	20	66	51.0	9.43
PROMIS satisfaction with roles and activities v2.0	4177	22	68	42.6	9.97	4190	22	68	42.4	9.78	8368	22	68	42.5	9.87
PROMIS social isolation v2.0	4177	31	80	47.5	9.60	4190	31	80	47.4	9.50	8368	31	80	47.4	9.55

PROMIS, Patient-Reported Outcome Measurement Information System.

Summarized in Table 1 are scores for the NRS pain intensity scale and for PROMIS measures. Self-reported 7-day average pain intensity for the full sample was 5.6, a level interpreted as “moderate pain” when pain is categorized as mild, moderate, and severe.^15^ However, 35.5% of patients had “severe pain” defined as pain of 7 or greater. On average, patients' symptoms and function were worse compared with the PROMIS general US population on which the scores were centered.^17^ Patients in the sample reported depression and anxiety symptoms that were approximately a half standard deviation greater than those of the PROMIS reference sample and fatigue that was almost a full standard deviation higher. The biggest differences compared with the PROMIS reference sample, however, were for physical function and pain interference whose means in the full sample were 36.9 and 64.5, respectively.

#### 3.1.1. Interitem consistency

Cronbach's alpha value for the total PCA scale was 0.944. For the helplessness, magnification, and rumination subscales, values were 0.901, 0.760, and 0.913 respectively.

### 3.2. Factor analyses

The EFA, CFA, and bifactor results were virtually identical when conducted on Sample_MODEL_ and Sample_X-VALID_. For example, the greatest differences in factor loadings in EFA, CFA_DIM=1_, and CFA_DIM=3_, and bifactor loadings between samples was 0.062, 0.019, 0.016, 0.035, respectively. To conserve space, the results for Sample_X-VALID_ are not shown but are available from the corresponding author.

#### 3.2.1. Exploratory factor analyses results

Table 2 reports the findings from the EFAs. The factor loadings for a single factor ranged from 0.673 (“I keep thinking of other painful events,” magnification subscale) to 0.900 (“I keep thinking about how much it hurts,” rumination subscale). The first and second eigenvalues were 8.610 and 0.761 (ratio of 11.3). The first factor accounted for 66.2% of the variance. These results support a single factor solution.

**Table 2:** Exploratory factor analyses item loadings for helplessness (HELP), magnification (MAG), and rumination (RUM) questions (Q); based on modeling sample; one and three factors extracted.

One factor loadings		Three factor loadings		
Item	1	1	2	3
Q_1_HELP	0.770	0.412	0.292	0.151
Q_2_HELP	0.753	0.706	−0.037	0.152
Q_3_HELP	0.814	0.647	0.101	0.145
Q_4_HELP	0.874	0.800	0.167	−0.02
Q_5_HELP	0.859	0.799	0.118	0.012
Q_12_HEL	0.756	0.21	0.377	0.261
Q_6_MAG	0.772	0.257	0.112	0.512
Q_7_MAG	0.673	0.189	−0.004	0.581
Q_13_MAG	0.689	−0.086	0.019	0.882
Q_8_RUM	0.801	0.096	0.577	0.214
Q_9_RUM	0.866	0.121	0.716	0.103
Q_10_RUM	0.900	0.07	0.876	0.015
Q_11_RUM	0.899	0.134	0.780	0.051

Q1-HELP, I worry all the time about whether the pain will end; Q2-HELP, I feel I cannot go on; Q3-HELP:it is terrible and I think it is never going to get any better; Q4-HELP- it is awful and I feel that it overwhelms me; Q5-HELP, I feel I cannot stand it anymore; Q6-MAG, I become afraid that the pain will get worse; Q7-MAG, I keep thinking of other painful events; Q8-RUM, I anxiously want the pain to go away; Q9-RUM, I cannot seem to keep it out of my mind; Q10-RUM, I keep thinking about how much it hurts; Q11-RUM, I keep thinking about how badly I want the pain to stop; Q12-HELP, there is nothing I can do to reduce the intensity of the pain; Q13-MAG, I wonder whether something serious may happen.

In a follow-up EFA, we extracted a 3-factor solution to explore the putative 3-factor structure of PCA responses. These results are also reported in Table 2. The loadings aligned with the hypothesized structure of the PCS except for 1 item, “there is nothing I can do to reduce the intensity of the pain”—an item from the helplessness subscale, which loaded below 0.40, and was more highly associated with the rumination items (0.377) than with the helplessness items (0.210). Intercorrelations among the 3 factors were high. Correlations with Factor 1 for Factors 2 and 3 were 0.717 and 0.742, respectively; the correlation between Factors 2 and 3 was 0.701.

#### 3.2.2. Confirmatory factor analyses results

Table 3 compares the results from the 2 CFA models in which a single factor and 3 factors are hypothesized (CFA_DIM=1_ and CFA_DIM=3_, respectively). Comparative fit index, TLI, and RMSEA values for CFA_DIM=1_ model were 0.965, 0.957, and 0.136, respectively. For the CFA_DIM=3_, the values of CFI, TLI, and RMSEA were better (0.986, 0.983, and 0.084, respectively), but at the cost of parsimony.

**Table 3:** One- and 2-dimensional confirmatory factor analysis (CFA) results (based on the modeling sample).

	1 factor (CFA_DIM = 1_)		3 factor (CFA_DIM = 3_)					
	1		1		2		3	
	Loading	SE	Loading	SE	Loading	SE	Loading	SE
Q_1_HELP	0.770	0.007	0.794	0.007				
Q_2_HELP	0.753	0.008	0.769	0.008				
Q_3_HELP	0.814	0.006	0.832	0.006				
Q_12_HELP	0.756	0.007	0.896	0.004				
Q_4_HELP	0.874	0.004	0.878	0.004				
Q_5_HELP	0.859	0.005	0.781	0.007				
Q_6_MAG	0.772	0.006			0.855	0.007		
Q_7_MAG	0.673	0.011			0.733	0.012		
Q_13_MAG	0.689	0.009			0.753	0.009		
Q_8_RUM	0.801	0.006					0.829	0.006
Q_9_RUM	0.866	0.004					0.891	0.004
Q_10_RUM	0.900	0.003					0.919	0.003
Q_11_RUM	0.899	0.003					0.922	0.003

Q1-HELP, I worry all the time about whether the pain will end; Q2-HELP, I feel I cannot go on; Q3-HELP, it is terrible and I think it is never going to get any better; Q4-HELP- it is awful and I feel that it overwhelms me; Q5-HELP, I feel I cannot stand it anymore; Q6-MAG, I become afraid that the pain will get worse; Q7-MAG, I keep thinking of other painful events; Q8-RUM, I anxiously want the pain to go away; Q9-RUM, I cannot seem to keep it out of my mind; Q10-RUM, I keep thinking about how much it hurts; Q11-RUM, I keep thinking about how badly I want the pain to stop; Q12-HELP, there is nothing I can do to reduce the intensity of the pain; Q13-MAG, I wonder whether something serious may happen.

#### 3.2.3. Bifactor results

Finally, we fit a bifactor model in which all items loaded on a general factor and, in addition, loaded on a specific factor identified based on their subscale designations. Comparative fit index, TLI, and RMSEA values for the bifactor model were 0.993, 0.989, and 0.069, respectively. Table 4 displays the general and group factor loadings for the bifactor model. For ease of comparison, the CFA_DIM=1_ factor loadings are replicated in the table. The most salient finding is the degree of similarity between the factor loadings in the unidimensional CFA model and those for the general factor of the bifactor model (largest difference = 0.076). Larger variations would be expected when data have substantial multidimensionality. Also of note is the fact that the HELP subscale item, “there is nothing I can do to reduce the intensity of the pain” had a low negative loading on the HELP factor after the variance of the general factor was extracted; that is, the item accounted for no additional reliability once the general factor variance was accounted for. Bifactor statistics also confirmed the essential unidimensionality of the data. Omega-H was 0.97 for both Sample_MODEL_ and Sample_VALID_, well above the recommended criterion of >0.8 for confirming a measure's essential unidimensionality.^30^ Once the general factor was accounted for, the subscales accounted only for small amounts of variance. Omega-subscale values for the helplessness, magnification, and rumination subscales were 0.06, 0.00, and 0.014, respectively in the Sample_MODEL_ and 0.08, 0.00, and 0.17 for Sample_VALID_. Explained common variance of the general factor was 0.96 for both samples, well above the recommended criterion of 0.90, and suggesting that subscale scores do not add value.

**Table 4:** Unidimensional and bifactor confirmatory factor item loadings for helplessness (HELP), magnification (MAG), and rumination (RUM) questions (Q); based on the modeling sample.

Items	Unidimensional	Bifactor			
	One-factor	General factor	Helplessness	Magnification	Rumination
	Loading	Loading	Loading	Loading	Loading
Q_1_HELP	0.770	0.787	0.062		
Q_2_HELP	0.753	0.722	0.330		
Q_3_HELP	0.814	0.804	0.215		
Q_4_HELP	0.874	0.850	0.330		
Q_5_HELP	0.859	0.828	0.353		
Q_12_HELP	0.756	0.795	−0.090		
Q_6_MAG	0.772	0.782		0.236	
Q_7_MAG	0.673	0.668		0.291	
Q_13_MAG	0.689	0.683		0.462	
Q_8_RUM	0.801	0.779			0.237
Q_9_RUM	0.866	0.819			0.335
Q_10_RUM	0.900	0.824			0.462
Q_11_RUM	0.899	0.844			0.357

Q1-HELP, I worry all the time about whether the pain will end; Q2-HELP, I feel I cannot go on; Q3-HELP, it is terrible and I think it is never going to get any better; Q4-HELP- it is awful and I feel that it overwhelms me; Q5-HELP, I feel I cannot stand it anymore; Q6-MAG, I become afraid that the pain will get worse; Q7-MAG, I keep thinking of other painful events; Q8-RUM, I anxiously want the pain to go away; Q9-RUM, I cannot seem to keep it out of my mind; Q10-RUM, I keep thinking about how much it hurts; Q11-RUM, I keep thinking about how badly I want the pain to stop; Q12-HELP, there is nothing I can do to reduce the intensity of the pain; Q13-MAG, I wonder whether something serious may happen.

## 4. Discussion and conclusions

The results of this study provide substantial evidence for the essential unidimensionality of the PCS total scores. The EFA results did not warrant extracting more than one factor. When 3 factors were forced, the results aligned with the putative subscale structure with the notable exception of 1 item from the helplessness subscale that loaded 0.37 on rumination and only 0.210 with its designated subscale. The fit of a CFA model that posited a single factor was acceptable. A 3 dimensional model had better fit, but at the cost of parsimony.

The bifactor results were the most telling because they quantified the reliability of the subscale scores after a general factor was extracted. Whereas the reliability (omega-H) for the general factor was above 0.96, the reliability of the subscales was 0.14 and 0.17 at its highest (rumination subscale) and was 0.00 for magnification subscale scores.

A limitation of this study is the demographic homogeneity of the sample. Future studies could attempt to replicate the findings with a more racially and ethnically diverse sample. However, a number of factors support confidence in the findings. First, the sample was large and comprised of patients with heterogeneous chronic pain problems seeking care from a large pain management center, and thus, the variable of interest—pain catastrophizing—was relevant to respondents. Second, the consistency in results across the model and validity samples indicates that the results are likely to generalize to other populations of people with chronic pain. Finally, the use of bifactor analyses allowed quantification of what subscale scores add beyond the general factor scores.

Two published studies had findings that seem to conflict with our results. Craner et al.^10^ applied hierarchical multiple regression analyses to evaluate the contributions of PCS subscale scores in accounting for pain and quality of life variables. When the subscales were entered as individual predictors, the helplessness and magnification scores, but not rumination scores, accounted for unique variance in several tested variables. In another study, Gilliam et al. tested the mediating impact of PCS subscale scores in treatment outcomes.^12^ Improvement in helplessness scores proved to be the most consistent mediator in treatment outcomes. Magnification subscale scores had the least mediating effect. The findings of both of these studies highlighted potential weakness of the magnification subscale in comparison with the other subscales. In our study, the magnification subscale had the lowest interitem consistency of the subscales (alpha = 0.760 in the full sample) and the lowest subscale reliability after the general factor was extracted (0.00 in both model and validation samples). Future research should evaluate whether these findings are replicable.

Joint efforts between theory development and measurement science could further distinguish the clinical relevance of the PCS subscales in characterizing the impact of pain catastrophizing in individuals' pain experiences. Our findings did not support added value in subscale scores. From a measurement perspective, there seems to be little difference in what is being measured by the subscale items and what is being measured by the full scale. However, It is possible that these subdomains are clinically meaningful, but the PCS (at least as presently constructed) fails to reliably discriminate these distinctions. The clinically relevant question is whether the distinctions fail only at the measurement level or do they also fail at the theoretical level. One possibility is that the differences found in previous studies, although attributed to different pain catastrophizing domains,^10,12^ actually are reflective of differences in the ability of individual items to discriminate among respondents. If one or more PCS subscale is composed of items with better psychometric properties (eg, items with higher discrimination), then it would not be surprising to find the subscale comprised of the better items would be more responsive to change and more highly correlated with clinical anchors. This possibility could be evaluated by modeling the PCS item responses using an IRT model that estimates both item difficulty (intensity of the item) and item discrimination.

Our findings have implications for how the PCS is administered. If PCS item responses meet the other assumptions of a unidimensional IRT model, the PCS could comprise a calibrated item bank, and the items could be administered using CAT. In settings in which CAT is not feasible, a short form version of the PCS could be constructed based on IRT modeling. Reducing the burden of measuring pain catastrophizing could increase its use in clinical settings, providing clinicians with greater understanding of the psychosocial context of their patients' pain. However, a study that calibrated PCS responses using a one parameter IRT model (Rasch model), found, for 2 items, disordered category responses; for example, a response of “3” was associated with higher levels of catastrophizing than a response of “2.”^41^

Recently, a new measure of pain catastrophizing was developed by Amtmann et al.,^1^ the Concerns About Pain (CAP) scale. This measure was developed using IRT analysis and can be administered using CAT or one of 3 short forms (2-items, 6-items, and 8-items). Development of the scale included extensive qualitative analyses,^2^ the full bank has a reading level of 3.4, and a crosswalk is available to associate CAP scores with PCS scores. With these advances, researchers can consider both the CAP and the PCS for measuring pain catastrophizing.

## Disclosures

The authors have no conflicts of interest to declare.
